# Epithelial and stromal remodelling following femtosecond laser–assisted stromal lenticule addition keratoplasty (SLAK) for keratoconus

**DOI:** 10.1038/s41598-021-81626-5

**Published:** 2021-01-27

**Authors:** Mario Nubile, Niccolò Salgari, Jodhbir S. Mehta, Roberta Calienno, Emanuele Erroi, Jessica Bondì, Manuela Lanzini, Yu-Chi Liu, Leonardo Mastropasqua

**Affiliations:** 1 Department of Medicine and Sciences of Ageing, Ophthalmic Clinic, University “G. D’Annunzio” of Chieti-Pescara, Via dei Vestini, 66100 Chieti, Italy; 2 Singapore National Eye Centre, Singapore, Singapore

**Keywords:** Translational research, Tissue engineering, Visual system

## Abstract

The purpose of this study was to evaluate corneal epithelium and stromal remodelling with anterior segment optical coherence tomography in patients who have undergone stromal lenticule addition keratoplasty (SLAK) for advanced keratoconus. This was a prospective non-comparative observational study. Fifteen eyes of 15 patients with advanced keratoconus underwent implantation with a cadaveric, donor negative meniscus-shaped intrastromal lenticule, produced with a femtosecond laser, into a stromal pocket dissected in the recipient cornea at a depth of 120 μm. Simulated keratometry, central corneal thickness (CTT), corneal thinnest point (CTP), central epithelial thickness (CET), central and peripheral lenticule thickness, anterior and posterior stromal thickness were measured. Regional central corneal epithelial thickness (CET) and variations in the inner annular area (IAT) and outer annular area (OAT) were also analysed. All parameters were measured preoperatively and 1, 3, and 6 months postoperatively. The average anterior Sim-k decreased from 59.63 ± 7.58 preoperatively to 57.19 ± 6.33 D 6 months postoperatively. CCT, CTP, CET, and OAT increased and IAT decreased significantly after 1 month. All parameters appeared unchanged at 6-months except that of OAT that further increased. Lenticule thickness was stable. In conclusion we observed that SLAK reshapes the cornea by central flattening with stromal thickening and epithelial thickness restoration.

## Introduction

Keratoconus is an asymmetric corneal ectatic disorder characterized by progressive corneal protrusion and thinning, causing irregular astigmatism and impaired visual function^1^. Conservative management involves the use of spectacles or rigid gas permeable contact lenses. If the condition progresses and patients become intolerant to contact lenses, surgical treatment with one of the several keratoplasty procedures available may be the only option for visual rehabilitation^2^. Despite good long-term graft survival, conventional corneal transplantation can be associated with many complications that will affect the final result^3^. These complications include graft rejection, infection, wound dehiscence, or high postoperative astigmatism. Also, it must be performed in a specialized centre with highly trained corneal surgeons, thus limiting its availability.

Small incision lenticule extraction (SMILE), is a refractive surgical technique that was first described by Sekundo et al. for the treatment of myopia and myopic astigmatism^4^. It involves the creation of an intrastromal lenticule and a peripheral incision, in an all in one procedure, using a femtosecond laser (FSL), followed by extraction of the lenticule. More recently there has been considerable interest in the re-implantation of the extracted lenticule^5^. Following preclinical studies in rabbits and non-human primates, the concept of lenticule implantation was shown to be safe and effective^6^.

Human clinical trials were concordant with animal studies for safety and efficacy for the treatment of hyperopia, aphakia, and presbyopia^7–10^. These procedures fall under the unifying term of stromal keratophakia; i.e., stromal lenticule implantation^11^. Basically, a customizable stromal lenticule is first created with FSL, and then it is implanted intracorneally^11^. The clinical utility of lenticule re-implantation in refractive surgery has garnered significant interest for therapeutic indications, primarily in the form of stromal lenticule addition keratoplasty (SLAK)^12^.

The first use of this procedure for the correction of keratoconus was reported in 2018^13^. A negative meniscus shaped lenticule was first created from a donor cadaver cornea. This was then implanted into a hyperprolate recipient cornea of a keratoconus patient. Improvements in corneal curvature were achieved by inducing a flattening of the cone apex. Instead of subtracting or substituting the recipient tissue, corneal remodelling was obtained by the addition of stromal layers, that also produced the effect of thickening of the ectatic cornea^13^.

Epithelial and stromal remodelling following refractive surgery are important indicators for predicting the final clinical and refractive results^14–16^. This study aimed at investigating corneal epithelial and stromal remodelling following stromal lenticule addition keratoplasty (SLAK) using a high-resolution, spectral domain, anterior segment OCT (AS-OCT).

## Results

Fifteen consecutive patients affected by advanced keratoconus scheduled to undergo SLAK were enrolled in the present study. In three cases, familiar history of keratoconus was reported but the severity of the disease in the affected relatives was not known. Cases were defined as advanced based on topographic grading (grade 3–4)^17^, unsatisfactory visual acuity with spectacle correction, and contact lens intolerance. Mean age at surgery was 36 ± 11 years (range 18–52). Eleven patients were men and four women. All patients completed the 6-month follow-up. All surgeries were performed without intraoperative complications or relevant postoperative adverse events, except for mild and transient stromal oedema during the initial post-operative period. Overall, patients showed a mean improvement of corrected distance visual acuity from 1.07 ± 0.18 to 0.67 ± 0.22 LogMAR.

### Corneal topography and tomography

Nine patients were graded as stage 3 and six as stage 4 based on corneal topography^17^. Preoperative mean simulated keratometry (Sim-K) was 59.63 ± 7.58 D, mean CCT was 408 ± 59 μm, and mean CTP was 362 ± 67 μm. The thinnest point of the cornea was located centrally or displaced by less than 1.00 mm infero-temporal in all cases.

**Figure 1:**
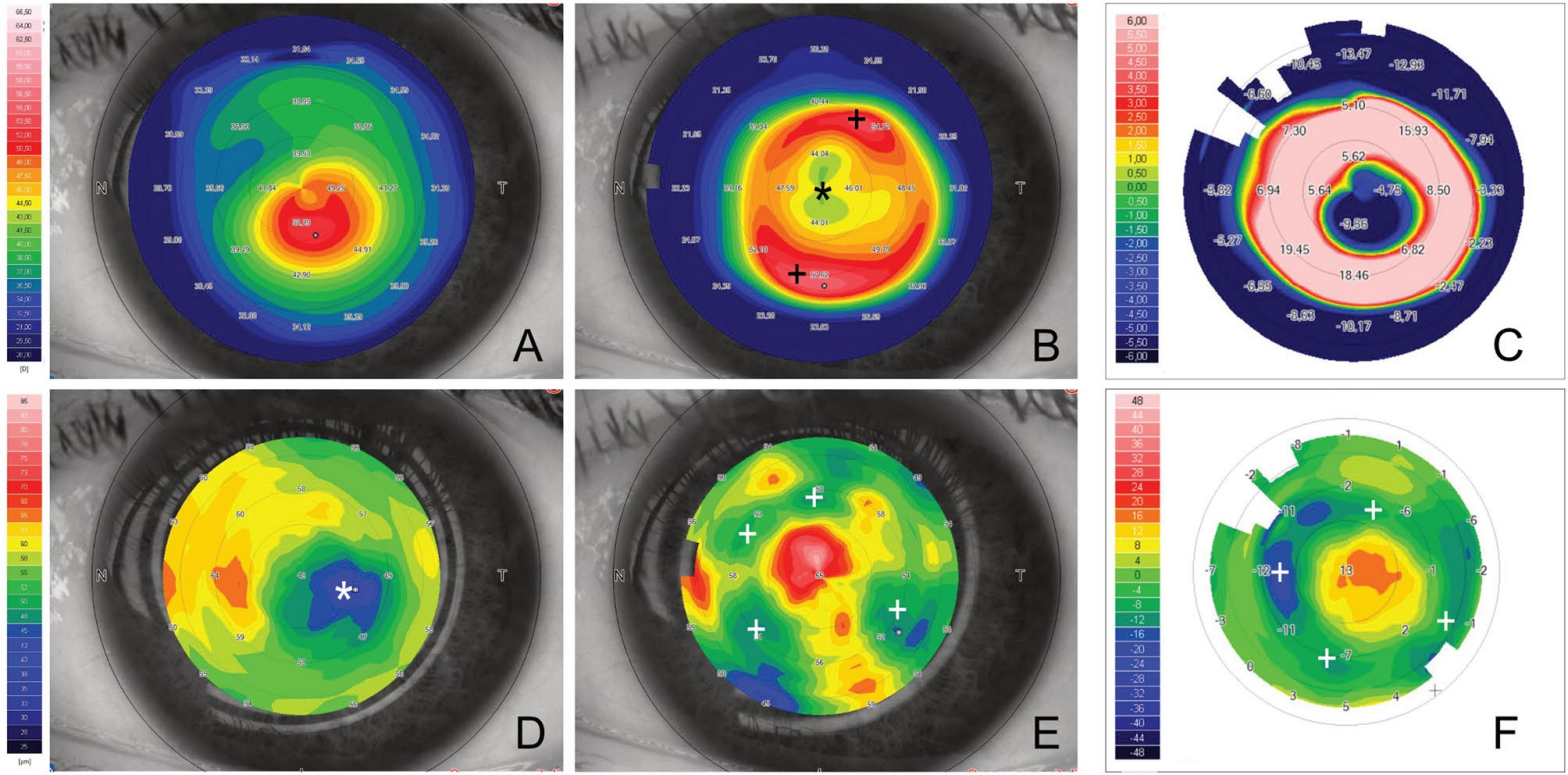
Anterior tangential curvature map of a keratoconus affected patient before (**A**) and 6 month after SLAK (**B**). The area of central flattening (black asterisk) is surrounded by a red ring of increased curvature corresponding to the transition zone of the lenticule (black plus signs). Differential map (**C**) showed an evident reduction of the curvature (blue color) of the cone apex, with an increase (red color) of mid-peripheral curvature. The flattened area corresponded to the cone apex. Preoperative epithelial map (**D**) showed thinnest point position corresponding to the cone apex (white asterisk). One month after SLAK (**E**), the epithelial thickness increased in the central area (central red isle) and moderately decreased in the areas of lenticule transition zone (white plus signs). Differential map (**F**) showed areas of reduction (blue color) and increase (yellow and orange) of the epithelial thickness compared to preoperative values. Notably the epithelial thickness increase corresponded to the apex of the cone, where the flattening effect was achieved.

At 1 month after surgery mean anterior Sim-K was 57.42 ± 6.88 D, with a mean reduction of − 2.21 D with respect to preoperative values (*p* < 0.01), while mean CCT and CTP were 472 ± 70 μm and 435 ± 68 μm respectively. Mean CCT increase was 64 ± 25 μm and mean CTP increase was 73 ± 27 μm with respect to preoperative values (*p* < 0.01). Anterior tangential curvature map revealed central flattening surrounded by an annular transition zone of increased curvature corresponding to the region of maximum thickness of the implanted lenticule (Fig. 1).

At 3- and 6-months follow-up mean anterior Sim-K was 57.59 ± 7.32 D and 57.19 ± 6.33 respectively, with no significant variations with respect to 1-month examination (*p* = 0.614). Similarly, CCT and CTP values at 3- and 6-months did not show significant changes over time with respect to 1 month (*p* = 0.529 and *p* = 0.689 respectively) (Table 1).

**Table 1:** Corneal curvature and thickness parameters before and 1, 3 and 6 months after SLAK.

	Curvature	Corneal parameters		Epithelial parameters			
	Sim-K	CCT	CTP	CET	ETP	IAT	OAT
Preoperative	59.63 ± 7.58	408 ± 59	362 ± 67	43 ± 5	31 ± 5	52 ± 4	56 ± 5
One month	57.42 ± 6.88	472 ± 70	435 ± 68	51 ± 7	38 ± 5	48 ± 3	61 ± 7
Three months	57.59 ± 7.32	476 ± 69	441 ± 74	50 ± 7	39 ± 6	49 ± 3	67 ± 6^†^
Six months	57.19 ± 6.33	475 ± 68	438 ± 73	50 ± 7	38 ± 6	49 ± 3	70 ± 6^‡^

*Sim-K* simulated keratometry, *CCT* central corneal thickness, *CTP* corneal thinnest point, *CET* central epithelial thickness, *ETP* epithelial thinnest point, *IAT* mean inner annular area thickness, *OAT* mean outer annular area thickness. Modification was statistically significance (*p* < 0.05) for all parameters with respect to preoperative. [Statistically significance (*p* < 0.05) variations were also reported between 3-month and 1-month† and 6-month and 3-months‡].

### Epithelial thickness

**Figure 2:**
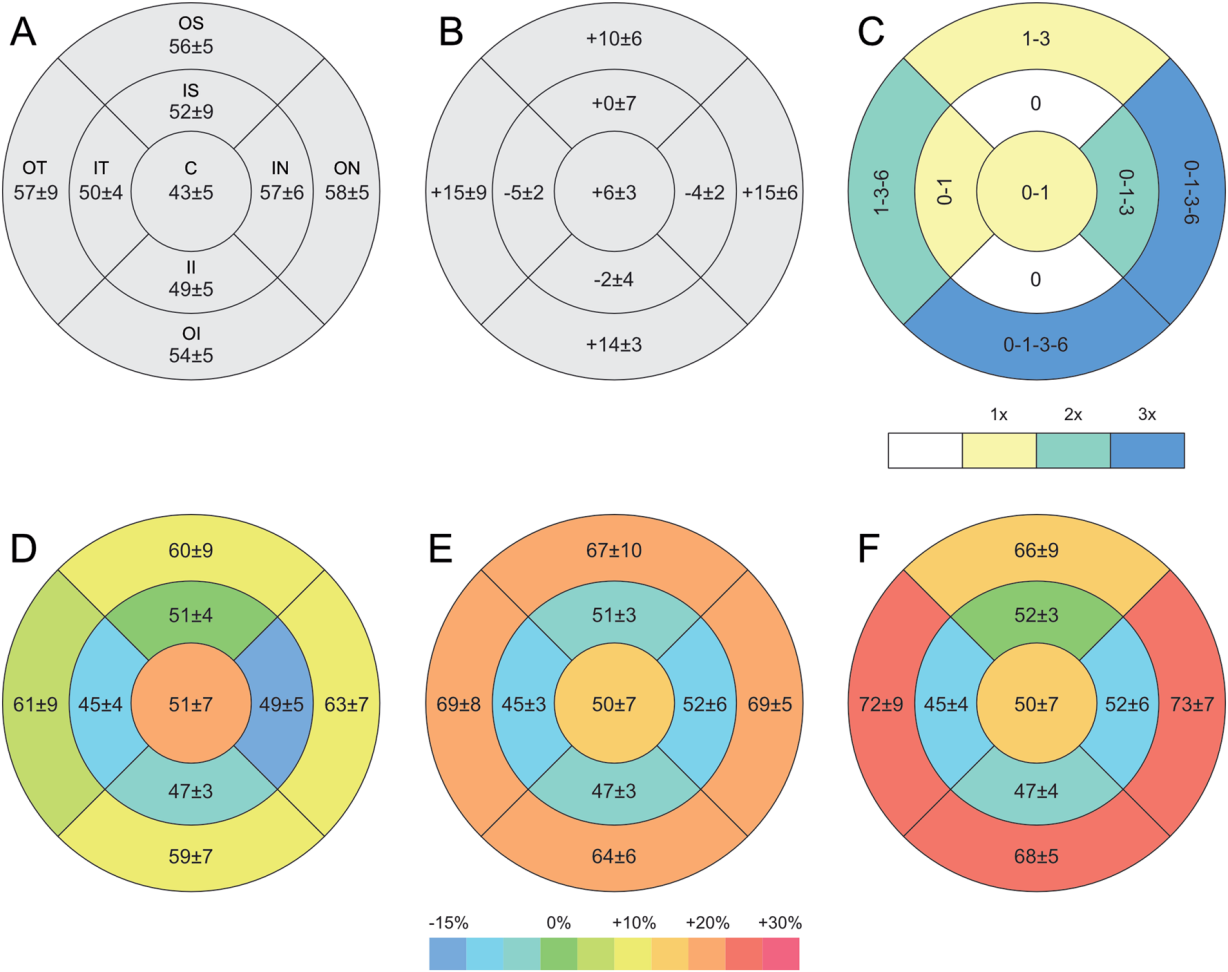
Epithelial thickness profile map with mean preoperative absolute values ± SD (**A**) and 6-month differences (**B**). Statistical significance changes of each sub-field and relative reference periods are reported in C with color-coding representing number of significant variations observed during the follow-up (white: no changes; 1×: change once; 2×: change at two time points; 3×: change at each time point). Mean value ± SD at 1 month (**D**), 3 months (**E**) and 6 months (**F**) are reported with color-coding representing percentage change from baseline (*C* central, *OS* outer-superior, *IS* inner-superior, *OT* outer-temporal, *IT* inner-temporal, *IN* inner-nasal, *ON* outer-nasal, *II* inner-inferior, *OI* outer-inferior).

The mean regional epithelial thickness parameters are reported in Table 1. Mean preoperative epithelial thickness was 43 ± 5 μm in the central area, 52 ± 4 μm in the inner annular area, and 56 ± 5 μm in the outer annular area. At 1 month, mean epithelial thickness change was + 7 ± 3 μm in the central area (*p* < 0.01), − 4 ± 2 μm in the inner and + 5 ± 2 μm in the outer annular areas (*p* = 0.013 and *p* = 0.04 respectively). Epithelial thickness map showed a central thickness increase and a mid-peripheral thickness reduction in the areas of annular increase of anterior corneal curvature (Fig. 1). The epithelial thickness map analysis showed a statistically significant change of all subfields with exception of the inner-superior and the inner-inferior subfields (Fig. 2).

**Figure 3:**
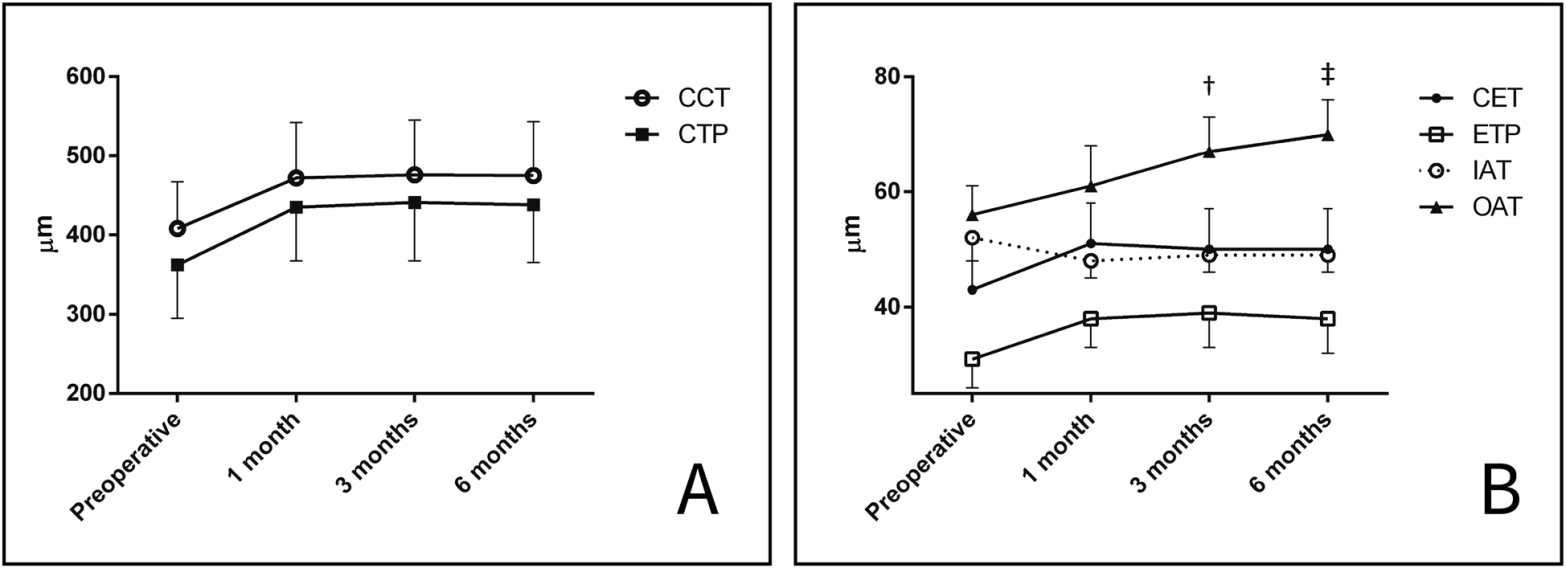
Corneal and epithelial thickness changes over time. Central corneal thickness (CCT) and Corneal thinnest point (CTP) significantly increased 1 month after surgery (panel **A**) and remained stable thereafter. Significant modifications of central epithelial thickness (CET), epithelial thinnest point (ETP), mean inner annular area thickness (IAT), and mean outer annular area thickness (OAT) were reported between preoperative and 1 month (panel **B**). OAT significantly increased also at 3 months (†) and 6 months(‡).

At 3-months, central epithelial thickness was 50 ± 7 μm (*p* = 0.832) while mean subfield thickness values of the inner annular area was 49 ± 3 μm (*p* = 0.286), with no significant changes as compared to 1-month. The mean outer annular area thickness significantly increased to 67 ± 6 μm (*p* < 0.01). Statistically significant variations of all the parameters were not observed after 6-months, except the outer annular area thickness that further increased to 70 ± 6 μm (*p* < 0.01). Six-month changes with respect to preoperative values were + 7 ± 3 µm, − 4 ± 2 µm, and + 14 ± 5 µm in central, inner, and outer areas, respectively. A graphical representation of epithelial and stromal thickness changes is presented in Fig. 3.

### Stromal remodelling

**Figure 4:**
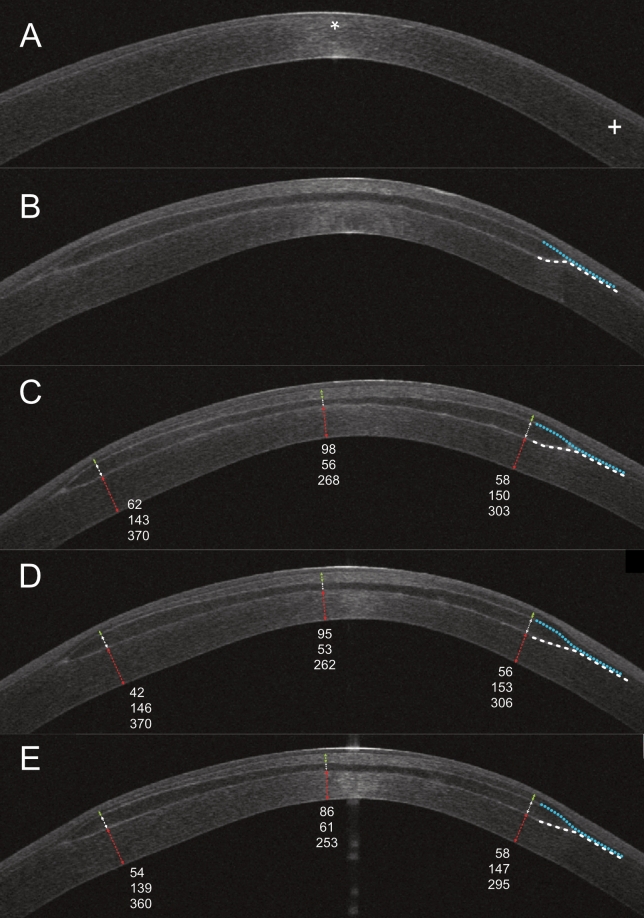
Preoperative AS-OCT horizontal scan of the central cornea (**A**) showed normo-reflective thinned stroma and reduced epithelial thickness in correspondence with the cone apex (white asterisk). 1 week after SLAK (**B**) the implanted lenticule appeared hypo-reflective in the central region with respect to the surrounding stroma and bordered by hyper-reflective interfaces. Anterior (green line), lenticule (white line) and posterior (red line) stromal thickness were measured in the center and at 3-mm radius. Measured thicknesses in microns are reported in the corresponding positions. Lenticule stroma remained hypo-reflective throughout the follow-up while lenticule wedge-shaped borders that were distorted by the transient tissue oedema at 1 week, regularized in shape over time with smoothening of transition zones of the lenticule, as marked by the dashed lines (**C**: 1 month, **D**: 3 months, **E**: 6 months).

High-resolution AS-OCT cross-sections allowed the identification of the intrastromal lenticule profiles in contrast to the surrounding recipient corneal stroma (Fig. 4).

Lenticule oedema was more prominent at the thickest part of the lenticule (i.e., the periphery) at one week after surgery. This caused a posterior displacement of the posterior stromal lamella. As the oedema resolved over time there was less distortion on the posterior lamella and more displacement of the anterior lamella caused by the volume of the additional stromal tissue. OCT imaging confirmed the precise lenticular profile inside the intrastromal pocket across the entire diameter, which corresponded to the lenticule shape before extraction.

At 1 month and throughout the follow-up, lenticules appeared hyporeflective in the central region and almost iso-reflective in the periphery with respect to recipient stroma. The mean measured central lenticule thickness was 21 μm thicker than expected (vs. 30 μm of programmed central thickness), while in the periphery the measured lenticule parameters were consistent with intended values. Mean central LT was 51 ± 15 μm (range 31–78) and mean peripheral LT was 150 ± 12 μm (range 138–161). The lenticule wedge-shaped edges were swollen 1 week after surgery but subsided during the first month, and no significant morphological changes were seen thereafter over the follow-up period. The donor–recipient interfaces were hyper-reflective at all time points. At 1-month postoperatively, posterior stromal reflectivity appeared higher in the region beneath the transition zone of the lenticule. The posterior reflectivity decreased over time but remained higher with respect to the surrounding recipient stroma up to 6 months. The anterior stromal reflectivity appeared unchanged throughout the follow-up. A representative case showing anterior segment OCT imaging of stromal changes before and after SLAK is presented in Fig. 4. Lenticule parameters at 3- and 6-months remained stable and did not show significant changes with respect to 1-month (Table 2).

**Table 2:** Anterior stromal thickness (AST), lenticule thickness (LT) and posterior stromal thickness (PST) at 1, 3 and 6 months after surgery in the central area and on 3-mm central radius (Mean ± SD).

	Central	Inferior	Superior	Nasal	Temporal
**One month**					
AST	86 ± 23	51 ± 11	59 ± 8	58 ± 7	65 ± 9
LT	51 ± 15	151 ± 9	143 ± 11	152 ± 16	152 ± 12
PST	286 ± 52	398 ± 56	426 ± 64	427 ± 47	369 ± 63
**Three months**					
AST	87 ± 11	52 ± 11	57 ± 10	56 ± 9	62 ± 11
LT	47 ± 9	151 ± 12	143 ± 15	149 ± 7	157 ± 11
PST	297 ± 56	401 ± 59	427 ± 62	428 ± 48	375 ± 68
**Six months**					
AST	84 ± 19	56 ± 8	61 ± 10	56 ± 7	65 ± 10
LT	49 ± 11	150 ± 11	139 ± 14	148 ± 7	152 ± 10
PST	295 ± 63	392 ± 49	424 ± 63	428 ± 51	368 ± 71

Statistically significant differences were not observed between the follow-up time points.

## Discussion

In advanced cases of keratoconus, corneal transplantation is indicated^3^. Alternative approaches to improve the central corneal curvature include the use of intracorneal ring segments (ICR)^21^ and, more recently, intrastromal lenticule transplantation^11,13,22,23^. ICR were shown to be effective in patients with moderate keratoconus but not in advanced cases^24^.

Stromal Lenticule Addition Keratoplasty (SLAK), a novel technique first described in 2018, reshapes hyperprolate pathological corneas avoiding the need for conventional lamellar or penetrating keratoplasty^12,13^. Reported outcomes include improved visual acuity (reduced myopic refractive error and manifest astigmatism) resulting from an improved the central corneal profile.

The average flattening of the anterior corneal curvature obtained in the patients treated in the present study (2.2 D of mean Sim-K reduction) was comparable with a previous report^13^. Other studies evaluated similar intrastromal additive keratoplasty techniques, differing in lenticule geometry and depth of implantation. Aliò et al. implanted a large isoplanar lenticule into keratoconic stroma obtaining comparable clinical results, with a mean anterior keratometry reduction from 1.58 to 2.00 D^22,23^. Both procedures aimed to stabilize the cornea and modify the corneal shape to improve visual acuity and quality.

High-resolution OCT imaging was shown to be useful to understand the intracorneal changes following ectatic disorders and in patients that have undergone refractive surgery^25^. Epithelial thinning at the corneal apex associated with epithelial thickening in the area around the apex was observed to be characteristic features in keratoconic patients in histological and in-vivo imaging, such as very high-frequency ultrasound, studies^26^. Studies using high-resolution AS-OCT confirmed these findings and were used to create a classification for the severity of keratoconus^27,28^. Anterior segment OCT combined with Placido topography (MS-39) used in this study was previously validated in normal eyes, keratoconic corneas, and eyes following excimer laser treatment^19,20^. In this study, high-resolution AS-OCT cross-sections allowed the identification of intrastromal lenticule profiles and the assessment of changes in the corneal epithelial and stromal regional thickness over time. Mild irregularities and swelling of the lenticule and recipient stroma, attributable to tissue edema, were detected only during the first month after surgery, and thereafter, the OCT scans confirmed minimal morphological changes over the follow-up period of 6 months.

These findings are in agreement with preliminary observations obtained using a different OCT system (RTVue, Optovue Inc., Fremont, CA)^13^. However, in the present study, an increase in stromal thickness that was consistent with the morphology and regional thickness variations of the lenticule profile was observed. Although the modifications of the stromal thickness induced by the implantation were consistent with the lenticule morphometry, both the CCT and CTP increases at 1 month were significantly higher (65 ± 28 μm and 67 ± 23 μm, respectively) than expected given the central lenticule thickness of 30 microns. The thickness remained stable over the 6-month follow-up.

The interpretation of the OCT analysis confirmed that this finding was due to several coexisting factors. The OCT measurements did include the lenticule interface boundaries since they were easily identifiable. Secondly, the measured values may be influenced by the changes in the recipient stromal layers adjacent to the lenticule interfaces. In fact, extracellular matrix remodelling, triggered by activated keratocytes, is likely to play a significant role in this sense, as documented by in vivo microscopic examination studies^29,30^ and animal studies after lenticule implantation for keratoconus^5,6^. Lastly, epithelial remodelling occurring after surgery contributing to the overall corneal thickness increase.

The thinned corneal epithelium at the cone apex significantly increased 1 month after surgery, stabilizing at 3-months, indicating that the remodelling might plateau sometime between 1 and 3 months. The epithelium has the capacity for significant remodelling in an attempt to regularize irregular curvatures^31,32^. The epithelial modifications were consistent with the normal corneal epithelial turnover; i.e., detectable as early as four weeks postoperatively. The flattening effect on the anterior corneal surface resulting from the lenticule implantation was associated with an epithelial remodelling that restored the central epithelial thickness values close to normality (increased from 43 to 50 microns, 15% gain). Corneal flattening following subtractive laser refractive surgery is thought to stimulate a significant increase in epithelial thickness^33^. The degree of thickening is related to the amount of surgically induced refractive correction^33^. Similarly, this study showed that the corneal flattening induced by additive as opposed to subtractive stromal techniques, were associated with epithelial remodelling with a significant thickening at the cone apex, evident at 1 month and stabilized at 3 months after surgery. Studies reporting corneal epithelial modifications occurring after ICR implantation in keratoconus are not available yet, and it would be interesting to assess whether this method of reshaping may exert similar modifications of the epithelium. However, studies reporting epithelial remodelling after CXL in keratoconus were characterized by a more uniform thickness distribution but not with a significant thickening, probably because the curvature changes induced by the crosslinking are negligible^34^.

Although the central modifications are important with respect to corneal optical quality, the results of this study also indicated that the epithelial remodelling occurred in the mid-peripheral and peripheral regions, with a slight decrease in the inner annular area (in the zones in which the thicker part of the lenticule produced a relative steepening), and a significant increase in the outer annular area. These findings were morphologically consistent with anterior corneal profile variations. The epithelial remodelling and thickness increase induced by SLAK may affect vision for different reasons. The regularization of the corneal epithelium could contribute to the reported improvement in best corrected VA^13,23^, and although the overall values for epithelial modifications were small, 7 microns represent at least 15% or more of the total epithelial thickness. Variations in epithelial thickness, in combination with the induced stromal flattening, directly affect the anterior curvature and the regularity of the surface of the cornea. Even small changes in the epithelium can effectively improve the dioptric power of the cornea as well as decrease higher-order aberrations (HOAs) by making the surface more regular^35^. Another possible advantage related to epithelial thickening in such eyes affected by advanced keratoconus is that this can improve contact lens tolerance and reduce the risk for epithelial breakdown or keratoconic ‘pips’. Patients with atrophic/thin epithelium were shown to have significantly reduced tolerance to contact lens wear compared to patients with intact or hypertrophic central epithelium^36^.

The stromal volume expansion effect at the cone apex, was related to the lenticule implantation itself and the secondary tissue healing response, in addition to the epithelial hyperplasia, to increase the CCT and CTP (+ 67 ± 21 and + 76 ± 26 microns, respectively). Some transient stromal oedema was observed in the first week after surgery, mainly affecting the posterior surface curvature, consistently with the lenticule profile, but, as demonstrated by our previous study that used confocal microscopy for stromal analysis^30^, it rapidly subsided after the first week (Fig. 4B). Fluid accumulation was not observed by confocal microscopy thereafter and this finding was consistent with the stable stromal reflectivity documented by OCT in the present study. Theoretically modifications of the recipient stromal extracellular matrix (ECM) may be possible due to its component reorganization after tissue addition and lamellae compaction, but specifically designed histo-pathological studies should be performed to investigate these changes. The overall effect was a significant increase in the thickness of the corneal thinnest point and this could be favourable when treating corneas with the thinnest point less than the values for which CXL is indicated. Other authors have proposed the use of a temporary ‘onlay’ refractive stromal lenticule during CXL procedure in thin corneas^37^. However, the concept of intrastromal permanent integration of lenticules offers wider and new possibilities for treatment. Not only can the patients safely undergo cross-linking, but after lenticule implantation they can also undergo sequential therapeutic excimer laser ablation. Although the cases included in our study were all non-progressive advanced keratoconus, it would be of interest to investigate whether stromal and epithelial expansion induced by SLAK could be performed concurrently with cross-linking techniques in progressive keratoconus.

This study was limited by a relatively small sample size and by limiting enrolment to patients affected with a central form of keratoconus. However, this is a new surgical technique hence restricting the selection to patients who theoretically should obtain maximum benefit, limiting the ability to evaluate overall remodelling in those patients with displaced cones which, biomechanically, maybe asymmetric in their tissue response. Moreover, the results were not compared with other keratoplasty techniques. Future studies should include evaluation of the in-vivo keratocyte-mediated wound healing response longitudinally at the lenticule interfaces^23^ and its effect on corneal biomechanics. In addition, with more cases, the complex epithelial and stromal remodelling mechanisms may differ with respect to preoperative keratoconus staging and morphology location of the cone apex, extent of stromal thinning, and geometry and depth of the intrastromal lenticule implant.

In conclusion, high-resolution AS-OCT evaluation of negative meniscus Intrastromal lenticules implantation in SLAK showed an induced corneal reshaping, characterized by central flattening of the cone apex associated with stromal thickening consistent with the shape of the implanted lenticule. However, stromal wound healing and epithelial hyperplasia contributed, over time to a greater extent of central corneal re-thickening. The maintenance of the transparency of the lenticule and its integration into the host cornea is critical for successful clinical outcomes, and both cellular response, structural and geometrical adaptation of the lenticule contributes to the therapeutic reshaping in a thin biomechanically weak keratoconic cornea.

## Patients and methods

### Patients

This prospective observational longitudinal study was approved by the University Institutional Review Board and adhered to the tenets of the Declaration of Helsinki. The setting of the trial was the Ophthalmology Clinic, Department of Medicine and Science of Ageing, University “G. d’Annunzio” of Chieti-Pescara, Italy. After a detailed explanation of the study, written informed consent was obtained from all patients.

The study enrolled fifteen consecutive eyes of 15 patients affected by stage 3–4 central keratoconus (according to the ABCD classification)^17^ that met the inclusion/exclusion criteria and treated with femtosecond laser–assisted Stromal Lenticule Addition Keratoplasty (SLAK), according to a previously described protocol^13^.

Inclusion criteria were corrected distance visual acuity (CDVA) worse than 20/200, contact lens intolerance, myopic refraction, apex of the cone within a 1.5-mm zone with steep keratometry between 53.00 and 70.00 D, and thinnest point equal or superior to 300 μm. Exclusion criteria were coexistence of any other ocular pathology such as dry eye or atopic keratoconjunctivitis and previous ocular surgeries.

### Surgical technique

Donor lenticules suitable for anterior lamellar keratoplasty according to a previously described protocol were obtained from human eye-bank donor cornea (Azienda Ospedaliera San Giovanni Addolorata, Rome, Italy)^12^. The mean donor age was 63.7 years old (range 51–69 years old). Negative serologic test results were obtained from all donors. The average death-to-enucleation time was 10 h (range 6–12 h) and the mean storage time (between eye bank procedures and femtosecond laser cut) was 27.5 h (range 23–48 h). Tissue was stored for at least 6 h in a dextran-enriched storage medium (Thin-C; Alchimia, Padova, Italy).

**Figure 5:**
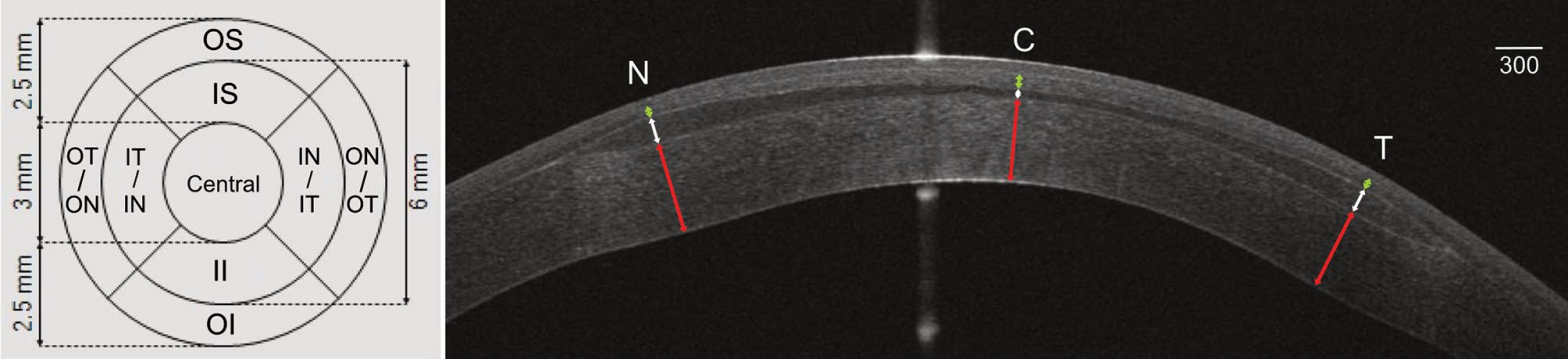
Epithelial sub-field map (left panel) with corresponding diameters and area acronyms (*C* central, *OS* outer superior, *IS* inner superior, *OT* outer temporal, *IT* inner temporal, *OI* outer inferior, *II*: inner inferior, *ON* outer nasal, *IN* inner nasal). Horizontal OCT scan (right panel) with representation of calliper positions used for measurement of posterior stromal thickness (PST: red arrows), lenticule thickness (LT: white arrows) and anterior stromal thickness (AST: green arrows), in the central and peripheral lenticule zones.

Corneal-scleral rims were mounted onto an artificial anterior chamber (Network Medical Products Ltd., Coronet House, United Kingdom) and underwent hyperopic FLEx (Femtosecond laser lenticule extraction) procedure using a 500-kHz VisuMax femtosecond laser (Carl Zeiss Meditec, Jena, Germany). The obtained lenticules were negative-meniscus shaped and consisted of two distinct portions: a central 6.00 mm width concave-shaped optical zone with a peripheral thickness of 148 µm that centripetally taper reaching 30 µm in the center, and a peripheral 0.7 mm transition zone (Fig. 5).

Recipient corneas underwent a modified femtosecond laser–assisted FLAP procedure called ‘Flocket’ where an isoplanar intrastromal pocket at 120 µm depth from the anterior surface is created^18^. Flap-hinge length was set to 21.70 mm to obtain a single 4 mm wide superior incision. The diameter was set to 8.20 mm and the incision angle to 100°. The energy cut index was standardized to 35 nJ and the spot separation to 5 µm. Residual tissue bridges were separated in the intrastromal pocket with a blunt spatula, and the lenticule was then separated from the donor cornea and implanted into the recipient pocket by using customised forceps (Janach, Como, Italy) and spatulas. Fine distention and centration were completed with a gentle external massage and confirmed immediately after the procedure with an AS-OCT scan. Postoperatively, all patients received topical ofloxacin 0.3% six times daily and dexamethasone 0.15% four times daily. After 2 weeks, topical antibiotics were discontinued and the topical steroid was tapered over 8 weeks.

### Corneal optical coherence tomography examination

All patients enrolled underwent corneal tomography using an MS-39 OCT (CSO, Costruzione Strumenti Oftalmici, Florence, Italy) preoperatively and after 1, 3, and 6 months postoperatively. MS-39 uses Spectral-domain OCT (SD-OCT) and Placido Disk corneal topography to obtain measurements from the anterior segment of the eye. The repeatability of corneal sub-layer measurements performed with this device in healthy eyes and corneas affected by keratoconus was previously reported^19,20^.

After auto-calibration, one Placido top-view image and a series of 25 SD-OCT radial scans were acquired. MS-39 uses a Super Luminescent Light Emitting Diode (SLED) light source at 845 nm, which provides an axial resolution of 3.5 µm, a transverse resolution of 35 µm, and a maximum depth of 7.5 mm for each section (16 mm × 7.5 mm). Each scan is composed of 1024 A-scans. Ring edges were automatically detected on keratoscopy so that elevation, slope, and curvature data of the anterior corneal surface could be derived by the arc-step with the conic curves algorithm. Profiles of the anterior cornea, posterior cornea, anterior lens, and iris were obtained from the SD-OCT scans. Data for the anterior surface from the Placido image and SD-OCT scans were merged using the proprietary software of the instrument. Measurements for internal structures (corneal thicknesses of the corneal sublayers: epithelium, anterior stroma, lenticule, and posterior stroma) were derived from the SD-OCT data.

In order to avoid the effect of potential diurnal variations, measurements were acquired between 9 and 12 am. All measurements were performed by an experienced examiner according to the guidelines provided by the manufacturer. After achieving central alignment and adjusting the focus, the patient was asked to blink immediately after which a measurement was performed once adequate eyelid opening was achieved. Examination quality was automatically checked by the instrument for adequate coverage and correct centration. Only correctly performed acquisitions were used for analysis. MS-39 automatically calculates epithelial thickness in the central 8.0 mm zone and the built-in software (Phoenix version 3.6.1) provided an epithelial thickness map. If epithelial edge detection was incorrect, manual adjustments were performed. In this study, the central epithelial thickness over an area of 3.0 mm of diameter and the peripheral thickness in two different annular areas at 3.0–6.0 mm (inner annular area) and 6.0–8.0 mm (outer annular area) were measured. These areas were further divided into nasal, temporal, superior, and inferior subfields (Fig. 5).

Average simulated keratometry in the central 3.0 mm area (Sim-k), central corneal thickness (CCT), and corneal thinnest point (CTP) were also evaluated. Measurements of the stromal thickness changes over time were evaluated on horizontal and vertical OCT scans with manual callipers (Fig. 5) to assess tissue remodelling. Three different regional stromal thicknesses were identified on each scan: posterior stromal thickness (PST: distance between endothelium and posterior lenticule-host interface), lenticule thickness (LT: distance between the two lenticule-host interfaces), and anterior stromal thickness (AST: distance between anterior lenticule-host interface and the Bowman’s membrane profile). These stromal values were obtained in the center of the lenticule and the thickest part of the lenticule (3.00 mm peripherally, at the edge of the optical zone), in the superior, inferior, temporal, and nasal directions. Lenticule-host interfaces were identified as hyper-reflective intrastromal lines bordering the lenticule tissue.

AS-OCT scans were also obtained in the early postoperative period (1 week after surgery) in all patients, but images were considered only for morphological evaluation of the lenticule-stromal geometry and were not used for quantitative analysis because the confounding effect of tissue oedema, typically observed in the first days after surgery. Data was analysed with SPSS (version 23.0; IBM Corporation, Armonk, NY). Given the number of patients enrolled in this study, non-parametric statistical tests (Wilcoxon signed-rank and Friedman tests, as appropriate) were used.
